# High-resolution spectral domain optical coherence tomography and fundus autofluorescence correlation in tubercular serpiginouslike choroiditis

**DOI:** 10.1007/s12348-011-0037-7

**Published:** 2011-08-17

**Authors:** Reema Bansal, Pandurang Kulkarni, Amod Gupta, Vishali Gupta, Mangat R. Dogra

**Affiliations:** 1 Advanced Eye Centre, Post Graduate Institute of Medical Education and Research, Chandigarh, India; 2 Department of Ophthalmology, Advanced Eye Centre, Post Graduate Institute of Medical Education and Research, Chandigarh, 160012 India

**Keywords:** Fundus autofluorescence, Spectral domain optical coherence tomography, Tubercular serpiginouslike choroiditis

## Abstract

**Objective:**

This study aims to describe changes in high-resolution spectral domain optical coherence tomography (SD-OCT) scans with simultaneous fundus autoflorescence (FAF) signals in tubercular serpiginouslike choroiditis (SLC).

**Methods:**

Simultaneous SD-OCT and FAF imaging of eyes affected with SLC from acute stage until resolution of lesions was obtained using Spectralis HRA+OCT system (Heidelberg Engineering, Heidelberg, Germany).

**Patients:**

Four eyes (three patients) with SLC were prospectively followed.

**Results:**

Acute lesions of SLC (diffusely hyperautofluorescent) corresponded to hyperreflective areas on SD-OCT involving the retinal pigment epithelium (RPE), photoreceptor outer segment tips (POST), inner segment–outer segment (IS/OS) junction, external limiting membrane (ELM), and outer nuclear layer (ONL) with a minimal distortion of inner retinal layers. There was no backscattering from inner choroid. During healing, lesions became discrete with a hypoautofluorescent border and predominant hyperautofluorescence centrally. The hyperreflective fuzzy areas on SD-OCT scans disappeared, and irregular, knobbly elevations of outer retinal layers appeared. The RPE, POST, IS/OS junction, and ELM could not be distinguished. The ONL appeared normal. The choroid showed an increased reflectance. As the lesions healed further over the next 3–6 months, they became predominantly hypoautofluorescent with loss of RPE, POST, IS/OS junction, and ELM in SD-OCT scan.

**Conclusion:**

The SD-OCT provided an insight into the ultrastructural changes in the outer retina during the course of acute SLC lesions. The changes on OCT correlated with abnormal FAF findings.

Serpiginous choroiditis is a progressive, chronic, recurrent inflammatory disease primarily affecting the inner choroid and retinal pigment epithelial (RPE) cell layer [1]. Serpiginouslike choroiditis (SLC) of presumed tubercular etiology is a distinct clinical entity that begins usually as multifocal choroiditis lesions that coalesce and progress in a serpiginoid pattern [2, 3]. While the choriocapillaries have been shown to be the most affected layer in serpiginous choroiditis (SC), the primary site of inflammation in SLC is not yet known and is at best speculated, presumably the choriocapillaries [4].

The simultaneous recordings of topographic and tomographic images by using a combination of scanning laser ophthalmoscopy and optical coherence tomography (OCT) have increased our understanding of the pathogenesis of various diseases of the retina and choroid [5]. The Spectralis HRA+OCT (Heidelberg Engineering, Heidelberg, Germany) is a novel multimodal imaging device that enables us to correlate confocal angiograms, fundus autofluorescence (FAF) images, and other imaging modes with the high-resolution spectral domain (SD)-OCT scans.

The role of OCT in patients with uveitis is complementary to the conventional fundus photography and fundus fluorescein angiography (FA). More recently, FAF has emerged as a very sensitive imaging modality to evaluate inflammatory disorders affecting the chorioretinal interface. The combined FAF and OCT signals have been effectively used in the evaluation of uveitic macular edema in terms of visual outcome [6]. The Spectralis HRA+OCT has been used in imaging the choroid in intraocular inflammation using the inverted scan technique.

We describe the changes in high-resolution SD-OCT scans that were simultaneously obtained with FAF signals on Spectralis HRA+OCT in four eyes of three patients with SLC who were followed from the stage of acute lesion to the healed stage over a period of 3–6 months.

## Methods

We prospectively followed four eyes of three patients with active SLC. The diagnosis of SLC was made in the presence of multifocal choroiditis lesions with central healing and active edges that were hyperautofluorescent in areas of active edges and hypoautofluorescent in healed areas and showed early hypofluorescence and late hyperfluorescence of active lesions on FA. The etiology was presumed to be tubercular if there was (a) corroborative evidence (such as a positive tuberculin skin test or QuantiFERON-TB Gold test), (b) exclusion of all other known causes of infectious uveitis except tuberculosis and noninfectious uveitic syndromes, and (c) a favorable therapeutic response to antitubercular therapy. All patients underwent chest radiography that was normal. Besides a complete clinical evaluation that included best-corrected visual acuity (BCVA), intraocular pressure (IOP), slit lamp biomicroscopic examination, and conventional imaging methods (digital photography and FA, when required, on Visupac 450 Plus, Carl Zeiss, Jena, Germany), these patients additionally underwent Spectralis HRA+OCT (Heidelberg Engineering, Heidelberg, Germany) imaging with simultaneously obtained FAF and OCT images at all follow-ups. The Spectralis system uses Heidelberg Eye Explorer Software Version 1.5.0 with Image Capture Module Version 1.1.0. An infrared fundus image was acquired parallel to OCT scan to ensure correct placement of image before acquiring FAF images simultaneous with OCT scans. The pupils were dilated before acquiring all scans. The Spectralis scans along with the FAF images were obtained before doing FA, using the 30° field-of-view mode. All images were recorded using the automatic real-time mode. The image acquisition was done by selecting the dense volume scan type over a scan angle of 20° or 30° and by adjusting the width/height of the OCT scan (depending upon the extent of the lesions in the fundus). We routinely perform dense volume scan with simultaneous FAF and SD-OCT mode. We use infrared mode to focus the fundus image, and once focused, we switch to combined FAF and SD-OCT mode and capture the images simultaneously. An integrated eye tracking allowed for live averaging of FAF images and the SD-OCT scans. The baseline FAF-OCT images were defined as the reference images to enable acquisition of images at the same site during follow-up visits. Patients were seen every 2 weeks until the lesions healed. In addition to oral corticosteroids, all patients received four-drug antitubercular therapy including isoniazid (5 mg/kg/day), rifampicin (450 mg/day if body weight was ≤50 kg and 600 mg/day if body weight was >50 kg), ethambutol (15 mg/kg/day), and pyrazinamide (25 to 30 mg/kg/day) initially for 3 to 4 months. Thereafter, rifampicin and isoniazid are used for another 9–14 months. Pyridoxine supplementation was given to all patients receiving antitubercular therapy until cessation of therapy. The corticosteroids were tapered depending upon the clinical response. All patients were receiving antitubercular therapy until their last visit and did not show any recurrence of inflammation.

Demographic details and treatment response to oral corticosteroids and antitubercular therapy were also noted. The OCT scans were analyzed and correlated with FAF/FA images in acute as well as healing stages.

## Results

During the course of the disease in patients with SLC, we observed a progressively changing pattern on SD-OCT scans that was consistent with the abnormal FAF signals detected simultaneously.

1. In an acute lesion of SLC, there was an ill-defined area of increased autofluorescence around the lesion. The SD-OCT passing through the area showed a localized, fuzzy area of hyperreflectivity in the outer retinal layers involving the RPE, photoreceptor outer segment tips (POST), photoreceptor inner segment–outer segment (IS/OS) junction, external limiting membrane (ELM), and the outer nuclear layer (ONL). The lesion was localized external to the outer plexiform layer with a mild distortion of the inner retinal layers. There was no increased backscattering from the inner choroid.
2. As the lesions started to heal, they became well defined and acquired a thin border of hypoautofluorescence while remaining predominant hyperautofluorescent centrally. The SD-OCT scan through the hyperautofluorescent area showed disappearance of the hyperreflective fuzzy areas that were replaced by irregular, hyperreflective knobbly elevations of the outer retinal layers. The RPE, the POST, IS/OS junction, and the ELM could not be distinguished. The ONL appeared normal. At this stage, there was an increased reflectance from the choroidal layers due to attenuating RPE–photoreceptor complex.
3. As the lesions healed further over the next 3–6 months, they appeared stippled with predominantly hypoautofluorescence. The SD-OCT scan showed loss of RPE, POST, IS/OS junction, and ELM. The increased reflectance from the choroid persisted.

**Fig. 1:**
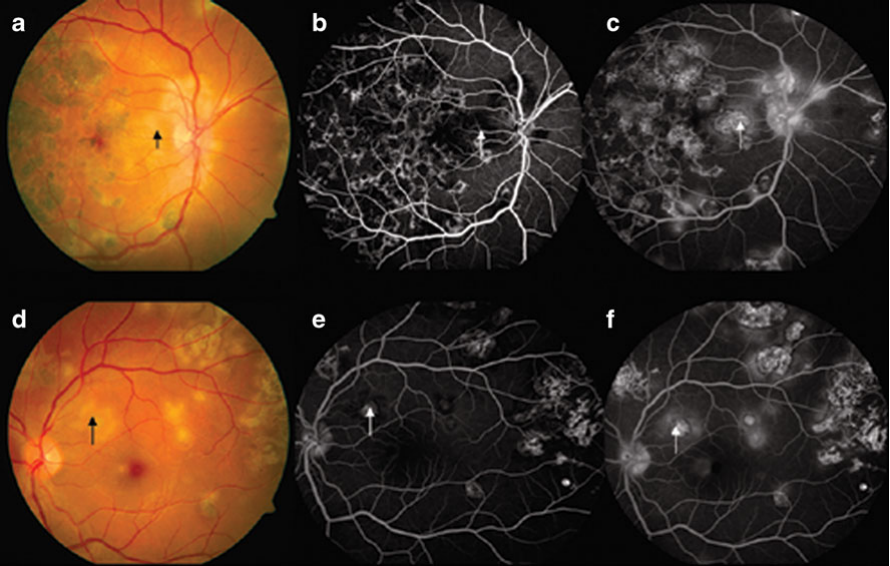
**a–f** Right eye fundus picture of patient #2 with inactive and active (*arrows*) lesions of serpiginouslike choroiditis (**a**) that appeared hypofluorescent in early (**b**) and hyperfluorescent in late-phase fluorescein angiogram (**c**). The left eye showed similar lesions (**d**–**f**)

**Fig. 2:**
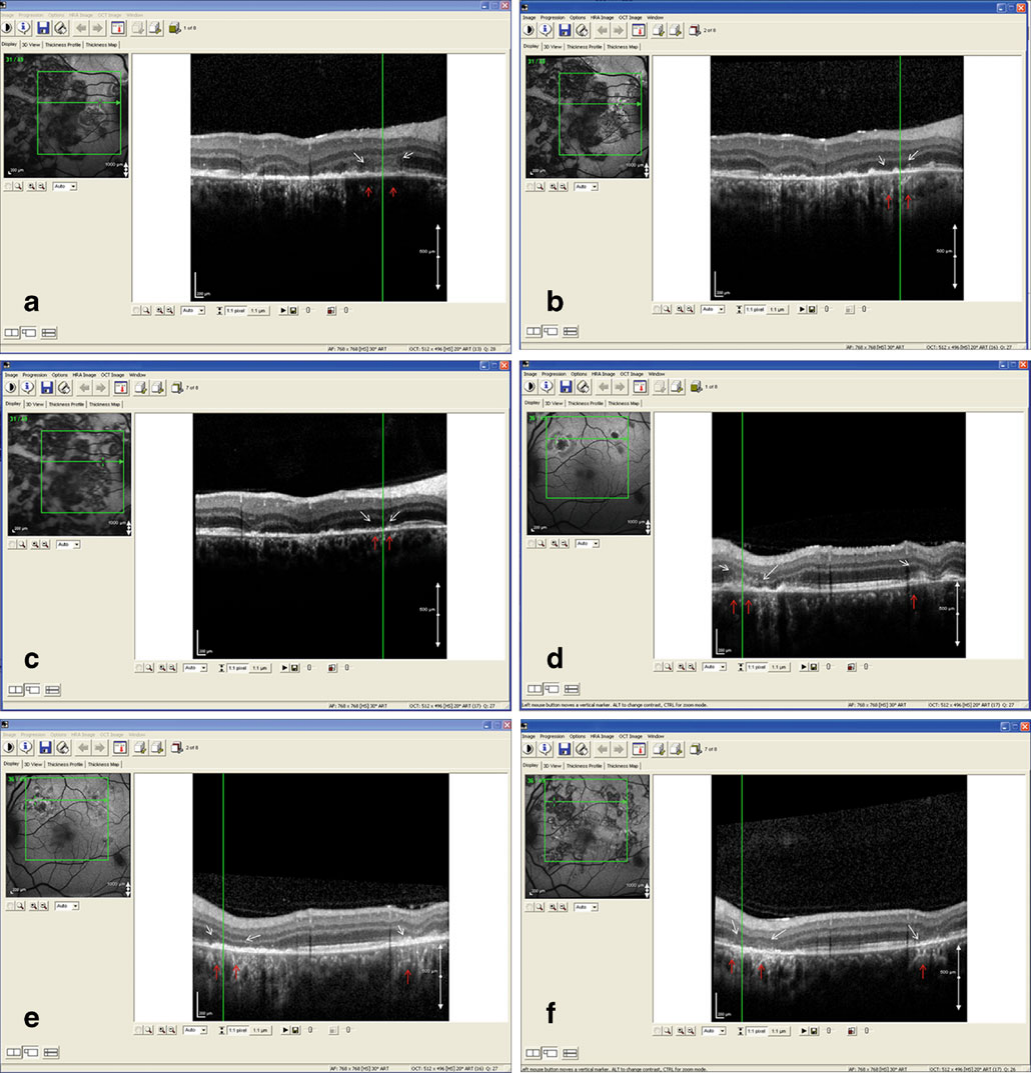
Combined fundus autofluorescence (FAF) and spectral domain optical coherence tomography (SD-OCT) images through the active lesion(s). The *green frame* (*left panel*) indicates the borders of the scanned area. The position marker corresponds to the retinal location through which the displayed OCT scan is obtained. In the right eye (**a**–**c**), in acute stage (**a**), there is an ill-defined area of increased autofluorescence (*left panel*) with fuzzy area of hyperreflectivity in outer retinal layers (*white arrows*) involving the retinal pigment epithelium (RPE), photoreceptor outer segment tips (POST), photoreceptor inner segment–outer segment (IS–OS) junction, external limiting membrane (ELM), and outer nuclear layer (ONL) (*right panel*). The inner retinal layers showed mild distortion. There was absence of any backscattering from the inner choroid (*red arrows*). About 2 weeks later (**b**), as the lesions started healing, they became well defined with a thin hypoautofluorescent border and predominantly hyperautofluorescence centrally in the right eye (*left panel*). The SD-OCT showed irregular knobby elevations (*white arrows*) of the outer retinal layers (*right panel*). The RPE, POST, IS–OS junction, and ELM could not be distinguished. There was an increased reflectance from the choroidal layers (*red arrows*) due to disappearing RPE–photoreceptor complex. Three months later (**c**), as the lesions healed further, they appeared stippled with predominantly hypoautofluorescence (*left panel*). The SD-OCT scan showed loss of RPE, POST, IS–OS junction, and ELM (*white arrows*) (*right panel*). The increased backscattering of the choroid persisted (*red arrows*). **d**–**f** Similar changes were seen in the left eye in acute (**d**), healing (**e**), and healed (**f**) stages of active lesions of SLC

The clinical details and the findings on FAF and SD-OCT during the healing of lesions are listed in Table 1. These changes are illustrated in Figs. 1 and 2.

**Table 1:** Clinical details of patients with active tubercular serpiginouslike choroiditis along with findings on combined fundus autofluorescence and Spectralis domain optical coherence tomography imaging as the lesions evolved from an acute stage up to healed stage

Patient	Sex	Age	Eye	Initial visual acuity	AS inflammation	Vitreous cells	Type of SLC lesions	TST	QuantiFERON-TB Gold test	FAF of acute lesion	SD-OCT of acute lesion	FAF of healing lesion	SD-OCT of healing lesion	FAF of healed lesion	SD-OCT of healed lesion	Follow-up (months)	Final visual acuity
1	M	20	Right	CF 1 ft	Nil	++	Placoid	Positive	ND	Diffuse, feeble hyperautofluorescent	Fuzzy, hyperreflective areas involving RPE, POST, photoreceptor IS–OS junction, ELM and ONL	Central hyperautofluorescent with hypoautofluorescent border	Irregular, knobbly elevations of outer retinal layers that are indistinct. The ONL appears normal.	Predominantly hypoautofluorescent	Loss of RPE, POST, IS–OS junction, and ELM	5	CF 1 m
2	M	19	Right	6/9	Nil	+	Multifocal	Negative	Positive							3	6/9
			Left	6/6	Nil	+	Multifocal										6/6
3	M	35	Left	6/9	Nil	Nil	Multifocal	Positive	ND							6	6/6

*AS* anterior segment, *SLC* serpiginouslike choroiditis, *TST* tuberculin skin test, *M* male, CF counting fingers, *ND* not done, *AF* autofluorescence, *SD-OCT* spectral domain optical coherence tomography, *RPE* retinal pigment epithelium, *POST* photoreceptor outer segment tips, *IS–OS junction* inner segment–outer segment junction, *ELM* external limiting membrane, *ONL* outer nuclear layer

### Case example

Patient 2: A 19-year-old male presented with decreased vision in both eyes since 3 months. On examination, the BCVA was 6/9 and 6/6 in the right and left eyes, respectively. The IOP were 14 and 12 mmHg in the right and left eyes, respectively. Both eyes showed unremarkable anterior segment and multifocal lesions of active as well as inactive choroiditis in the posterior pole (Fig. 1a–f). Simultaneous FAF and SD-OCT imaging of the right eye revealed findings as explained in the “Results” Section (1.) (Fig. 2a). The QuantiFERON-TB Gold test was positive. Other laboratory tests were normal. He received four-drug antitubercular therapy with oral corticosteroids. About 2 weeks later, the lesions started to heal and appeared as described in “Results” Section (2.) (Fig. 2b). Three months later, the lesions healed further and appeared as explained in “Results” Section (3.) (Fig. 2c). Likewise, left eye imaging also showed a similar pattern of FAF and SD-OCT changes during acute, healing, and healed stages of the lesions (Fig. 2d–f).

## Discussion

Tubercular SLC can be clinically differentiated from SC by the frequent presence of vitritis and multifocal choroiditis lesions in posterior pole and periphery, often sparing the juxtapapillary region [3]. The lesions in SC are, however, usually around the optic disk and spread contiguously to the macula. People with SLC are from areas endemic for tuberculosis, have positive uveitis work-up for tuberculosis, and respond favorably to antitubercular therapy with oral corticosteroids.

The main histological findings described in SC are atrophy of the choriocapillaries, the RPE, and the photoreceptors [4, 7]. While the choriocapillaries was reported to be the most affected layer that appeared acellular, the large choroidal vessels were unremarkable. Moderate, diffuse lymphocytic infiltration of the choroid has been reported, with predominant RPE atrophy. Occasionally, RPE hypertrophy has been seen correlating with areas of pigment clumping clinically [4, 7]. On the other hand, clinicopathological correlation in tubercular SLC is still not known. Clinically, SLC appears to involve primarily the inner choroid and the RPE. However, isolation of *Mycobacterium tuberculosis* from the RPE in an eye with tubercular panuveitis has strongly suggested preferential localization of the mycobacteria in the RPE, even in eyes with panuveitis or related intraocular inflammation, including multifocal choroiditis or serpiginouslike choroiditis [8].

High-speed, high-resolution OCT, by providing unprecedented details, has enhanced our understanding of the ultrastructure of the retina [9]. Distinct scattering bands correspond to photoreceptor IS/OS junction, photoreceptor outer segment tips, and the RPE and represent the thick scattering bands of outer retina [9]. The SD-OCT changes in healed scars of SLC have shown disruption of the outer retina at the site of scars with loss of junction between the inner and outer segments of photoreceptors and thinning of RPE/Bruch membrane complex and a correspondent increase in light reflectivity from the choroid [3]. Areas of thickening of RPE/Bruch membrane complex have also been shown in the regions of scars. However, there is no report of SD-OCT changes in any active and healing stages of SLC lesions.

All eyes with active lesions of SLC in our patients illustrate the progressive changes in the outer retinal layers on OCT scans that correlated with the FAF changes. The FAF images obtained simultaneously demonstrated the transition from initial hyperautofluorescent of acute lesions to predominant hypoautofluorescent in the healed stage. Absence of any demonstrable changes in the inner choroid during the active stage of the lesion on OCT scans may suggest a primary involvement of the RPE and not the choroid in tubercular SLC lesions. The FAF signals provide a strong clue to the status of RPE cells in various degenerative, inflammatory, and neoplastic disease processes. The FAF is increased (hyperautofluorescence) in the presence of increased metabolic activity of the RPE and decreased (hypoautofluorescence) when there is loss of the RPE.

We observed that the structural changes on OCT scans occurring during the course of SLC followed a stepwise orderly sequence, similar to those as seen on the FAF images. Increased autofluorescence in the acute lesions seen as diffuse, subtle, feeble hyperautofluorescent probably reflects retinal edema which was structurally evident as hyperreflectivity spreading into the outer retinal layers in the OCT scans of our patients. This possibly suggests cellular infiltration or extracellular fluid accumulation in these layers due to inflammation. As the lesions started healing, hyperautofluorescence decreased and hypoautofluorescence increased due to loss of RPE. The outer retinal layers on OCT scans of our patients showed attenuation and progressive loss in the affected areas as the lesions healed. The FAF becomes increasingly hypoautofluorescent which indicates severe damage to RPE and photoreceptors. This was seen as an irreversible, collective loss of the outer retinal layers involving the RPE, photoreceptor outer and inner segments, and the ELM in OCT scans.

Acute inflammatory lesions involving the RPE often cause a thickening at the level of RPE. It is believed that the lesions in SC arise deep in the retina, and the overlying retina appears edematous. The edema subsides as the lesions heal, and the RPE–choriocapillaries undergo atrophy. There is loss of photoreceptor–RPE complex with variable degrees of RPE hyperplasia. Yeh et al. have hypothesized that RPE may be the site of primary insult and hence, more severely damaged in presumed tuberculosis-associated serpiginouslike choroidopathy [10]. Increased autofluorescence in acute phase may be due to a number of factors. The size, number, or content of the fluorophores in the RPE cells may be altered by inflammation which increases the fluorophores content by inducing certain prooxidative pathways. Increased autofluorescence in other inflammatory conditions also such as White Dot Syndromes has been correlated with areas of RPE elevation on OCT image during active disease [10]. Once healed, the hypoautofluorescent areas showed resolution of RPE abnormalities on OCT scan.

The FAF abnormalities (hyperautofluorescent in active stage progressively becoming hypoautofluorescent in healed stage) have been well recognized (unpublished data). We observed that in the very initial stages of disease occurrence, when there was feeble hyperautofluorescence in the areas of new lesions, the OCT showed hyperreflectivity in the outer retinal layers that was fuzzy and ill defined. The choroid did not show any reflectance. This is an important OCT finding during the acute stage of SLC that may reflect the site of primary insult in SLC. However, SD-OCT technology is not yet able to image the choroid similarly to the retina, and hence, the absence of choroid changes on SD-OCT is not enough to definitely exclude its primary involvement in SLC.

Hyperreflectivity in the outer retinal layers in an active SC lesion is believed to be suggestive of acute inflammation involving deeper retinal and choroidal structures. From the OCT findings of our patients, we speculate that in an acute lesion of SLC, there is an increased metabolic activity caused by primary inflammation of the RPE cells. Release of inflammatory mediators into the retinal layers adjacent to the RPE causes a fuzzy, hyperreflective appearance on SD-OCT. Following an acute inflammatory episode, the RPE cells undergo hyperplasia and hypertrophy which is evident as hyperautofluorescence on FAF due to increased collection of lipofuscin. This corresponds to the localized, knobbly elevations of the outer retinal layers which represents clumping of the inflamed RPE cells. Once these damaged RPE cells undergo atrophy, there is an irreversible loss of photoreceptors giving rise to the loss of the outer retinal layers on OCT. The simultaneous increased hypoautofluorescence depicts the atrophied RPE cells. The late pigmentation of the retinal scar associated with RPE hypertrophy or hyperplasia also leads to a decreased FAF signal (especially when photoreceptors are disrupted), as can also be seen in Figs. 1 and 2. The baring of choroidal vessels in healed lesions of SC in contrast to their masking by hyperpigmentary changes of the RPE in SLC may also be due to entirely different entities affecting the inner choroid and the RPE cells, respectively.

The limitations of our study include a small number of cases and lack of clinicopathologic correlation. The outer retinal bands on SD-OCT, particularly IS/OS junction and POST, have not been so far correlated to the histological structures, and such correlation is still mostly presumed. However, the sequential ultrastructural changes in the outer retinal morphology on SD-OCT scans as seen in our patients provide important information that may add a new dimension in understanding the primary site of pathology in inflammatory conditions affecting the choroid and the RPE–photoreceptor complex.
